# Lattice Parameters and Lattice Energies of High-Pressure Polymorphs of Some Alkali Halides

**DOI:** 10.6028/jres.068A.009

**Published:** 1964-02-01

**Authors:** C. E. Weir, G. J. Piermarini

## Abstract

Lattice parameters of the high-pressure forms of the alkali halides were determined. The lattice parameters were used to compare the lattice energies of the NaCl and CsCl type structures at the transition pressure. An analysis of the effect of experimental uncertainties on the calculated lattice energies showed that in almost every instance the BornMayer theory adequately accounts for the lattice energy of the high-pressure structure.

## 1. Introduction

A continuing widespread interest in the alkali halides arises, at least in part, from the early success of Born and Mayer [1]^1^ in calculating the lattice energies of the ionic crystals and the subsequent experimental verification of the calculated values by means of the Born-Haber cycle [2]. The success of the Born-Mayer model applies to the normal structures of the alkali halides, i.e., NaCl type or face-centered cubic structure (fcc) for all alkali halides except CsCl, CsBr, and CsI which assume the CsCl type or simple cubic structure (sc). Certain alkali halides normally in the fcc structure undergo a polymorphic transition to the denser sc structure at elevated pressures and until recently the BornMayer model had been unable to account for the relative stability of the two structure types. In a recent report Tosi and Fumi [3] have shown that earlier treatments of the equilibrium between the two structure types were subject to errors arising principally from an incorrect evaluation of the repulsive energy at the elevated pressures. On reevaluating the repulsive energy they demonstrated that the lattice energies of the two polymorphs at the transition pressure differed by not much more than the *P*Δ*V* energy reported for the transition. It should be noted that on the basis of Bridgman’s data [4, 5] it has been concluded that the thermal energy involved in the transition is negligible on the scale of energies of present concern in the lattice energies. Therefore from the treatment of Tosi and Fumi it appears that the transition occurs between structures which are very nearly equivalent energetically. Of additional interest is the fact that according to Tosi and Fumi [3] the repulsive energy in the denser CsCl type structure is less than that of the corresponding NaCl type structure.

Tosi and Fumi employed lattice parameters derived from Bridgman’s *P–V* data on the alkali halides [6] for their calculations. Inasmuch as the lattice energies are very sensitive to the lattice constants, it was considered desirable to obtain direct experimental values for these constants to permit a more accurate evaluation of the lattice energies of the two structures. This report presents experimental values for the lattice constants of the high-pressure forms of nine of the alkali halides. The high-pressure transitions of CsF and KF are being reported for the first time. The corresponding transition in RbF, which apparently represented the first transition observed in an alkali fluoride, was reported recently [7]. From the measured lattice parameters of the high-pressure forms the lattice energies are then calculated by the method described by Tosi and Fumi [3].

Polymorphism in the alkali halides at elevated pressures was discovered by Slater [8] in RbBr and RbI. Bridgman subsequently studied these transitions in some detail [4] and observed the corresponding transition in RbCl. Later, Bridgman located transitions in KC1, KBr, and KI and reported the thermodynamic constants of the transitions [5]. The transition pressures are of the order of 5 kb for the rubidium halides and 20kb for the potassium halides with only a small variation in pressure with type of anion. Although it had been suspected that the transition involved transformation from the NaCl type to the CsCl type structure this was not confirmed until Jacobs [9] determined the structure of the high-pressure form of RbI by X-ray diffraction. Much later Jamieson [10] reported that the high-pressure form of KI was also the CsCl type structure. This was confirmed recently in this laboratory [11]. Vereschagin and Kabalkina [12] and Adams and Davis [13] have reported the parameters of high-pressure forms of RbCl and RbI. The lattice parameter of a high-pressure form of RbF has also been reported [7].

## 2. Experimental Method and Materials

The apparatus and experimental technique developed here for X-ray studies at high pressures have been described in detail [11] and will be outlined very briefly here. A collimated beam of Mo K*_α_* X-rays is diffracted by a thin layer of the material under study that is squeezed between flat parallel surfaces of two diamonds. The diffraction rings are recorded photographically and their diameters are measured on the film. The specimen-to-film distance is obtained from the measured diameters of the rings at 1 bar and the known X-ray diffraction patterns [14, 15]. The pressure on the material is calculated from the applied load divided by the smaller of the two diamond pressure areas.

During these experiments the camera [11] was modified by inserting a fixed diaphragm which permitted simultaneous exposure of opposite quadrants of the film. The filmholder was then rotated 90° and the other pair of quadrants exposed. In these studies the diffraction patterns of the high-pressure forms were obtained first and then the corresponding patterns at 1 bar were photographed in the adjacent quadrants. The two patterns appeared on the same film for comparison purposes. The order of the experiments was adopted because of the hygroscopicity of the alkali fluorides and it was considered necessary to verify the fact that the fluorides remained anhydrous during the experiment.

Both Bridgman [4, 5] and Jacobs [9] have emphasized the difficulties arising from hysteresis, sluggishness, etc., in the transitions of the alkali halides. For these reasons no effort was made to undertake measurements at the reported transition pressures. All data were obtained at fixed pressures at least 1 kb above the reported transition pressures. In the fluorides where transition points have not been reported previously, no attempt was made to locate the transition within narrow limits.

Most of the alkali halides studied were reagent grade materials of commercial origin. RbI, however, was prepared from Rb_2_CO_3_ with freshly distilled HI. The fluorides were formed from the carbonates or nitrates by successive treatments with HF. In addition, commercially available specimens of the fluorides were also used. The fluorides were so hygroscopic that it was found necessary to introduce the hot, freshly fused salt into the X-ray cell. This procedure was successful in eliminating problems arising from absorption of moisture as demonstrated by the fact that the X-ray diffraction patterns of the anhydrous fluorides were obtained at least 8 hr after introducing the material into the cell. The known X-ray diffraction patterns [14, 15] served to verify the identity and purity of these materials.

## 3. X-ray Diffraction Data

The results of the X-ray diffraction measurements on the high-pressure phases are given in table 1, which gives the observed *d*-spacings, the assigned indices, and the lattice constant for each material studied. The average lattice constant and the pressure to which the data apply are given for each material.

Each *d*-spacing of table 1 represents the average of at least five independent measurements on the same film. Additional experiments performed on each material yielded essentially the same *d*-spacings. The data tabulated pertain to a film selected for sharpness of lines, number of lines, etc. The average lattice spacings represent the numerical averages of the tabular data to three significant figures although in some instances somewhat greater precision is indicated by the data.

The *d*-spacing for every observed diffraction ring is reported in table 1, and it will be noted that the stronger diffraction rings of the fcc phases, i.e., the 200 and 220 reflections, generally persist at the higher pressures. The persistence of the low-pressure phases may be attributed to sluggishness in the transitions or to pressure gradients in the specimen.

As shown in table 1 all observed spacings not attributable to the fcc phase are readily indexed on the basis of the sc structure. In some instances, the lattice parameters are deduced from two diffraction rings only. It is believed that sufficient data are available, however, to eliminate any uncertainties in the expected structure of the high pressure polymorphs of the alkali halides and that the lattice parameters so determined are as reliable as the other data. The lattice parameters agree reasonably well with previously reported values [9, 12, 13] with one notable exception. The lattice parameter for the sc form of KI is somewhat smaller than the values reported by Jamieson [10] and in the preliminary report on the instrument used here [11]. Since construction of the camera, refinements have been made to improve the precision, and the present value is considered to be more reliable than the earlier one. Several independent experiments have been made with two different cameras which reproduce the present value within the experimental error.

These data appear to represent the first reported observation of the high-pressure CsCl type structure for KF and CsF. In each case, several independent experiments were performed on salts of different origin to check the transition as well as the lattice parameters. The appearance of the high-pressure form was also verified in all three fluorides by a microscopic method [16]. The transition pressures reported for the fluorides (see table 2) are considered to be only estimates because of uncertainties in the pressure calibration and strong evidence for sluggishness in the transitions. It will be noted that the diffraction data for KF and CsF are reported at 35 kb and 48 kb respectively while the transition pressures are estimated to be only 20 kb. In both cases the higher pressure was required to produce a sufficiently strong diffraction pattern for satisfactory measurement; the transition pressure was estimated to be 20 kb because the strongest ring of the high pressure form could be identified at 20 kb but not at 15 kb.

Bridgman previously studied all the alkali fluorides except RbF and observed no transitions [17, 18]. His failure to obtain the transition in KF may be attributed to the fact that he did not carry the material to sufficiently high pressures. A similar explanation does not apply in the case of CsF because Bridgman studied this material to 42 kb at room temperature and to 56 kb at 137 °C. The probable explanation for the discrepancies in the findings is that Bridgman’s specimen was not anhydrous. The transition reported here can be observed microscopically in the anhydrous salt but not in material exposed to the atmosphere for a very short time. In this connection it may be noted that if Bridgman’s data on CsF and KF were obtained on partly hydrated materials the volume changes of table 2 may be in serious error. In addition all calculations involving the *P–V* data will be subject to similar errors.

The changes in volume at the transition points calculated from the lattice parameters are tabulated in table 3 with the corresponding values obtained by Bridgman [5] from volumetric measurements. It will be noted that the lattice parameters of the fcc phases involve correction of the 1 bar values by means of Bridgman’s volume data. The agreement appears reasonably good for the rubidium halides, but not for the potassium halides. The discrepancy in the potassium halides may arise from the difficulties in estimating Δ*V* in the *P–V* studies on the potassium salts in which the transitions exhibit a 13 kg/cm^2^ wide [5] region of indifference—an interval of apparent coexistence of to phases in equilibrium. Under such circumstances it is believed that Bridgman’s estimates of Δ*V* would tend to be low. The values for KF, RbF, and CsF obtained in the present studies appear to be somewhat erratic. This result is probably attributable in part to the absence of reliable *P–V* data on the flourides and in part to uncertainties in the transition pressures themselves.

## 4. Lattice Energies

### 4.1. Method of Calculation

Because Tosi and Fumi [3] have demonstrated that the repulsive parameters may not be considered independent of volume or structure in the alkali halides, they must be calculated by the method devised originally by Born and Mayer [1].

The first two derivatives of the internal energy are respectively,

$${\left(\partial U/\partial V\right)}_{T}=T_{\alpha }/\beta -P$$ (1)

and

$${\left(\partial^{2}U/\partial V^{2}\right)}_{T}=\left[1+\left\{{\left(\partial \beta /\partial T\right)}_{P}+{\left(\partial \beta /\partial P\right)}_{T}\alpha /\beta \right\}T/\beta \right]/V\beta$$ (2)

where, in addition to the usual quantities, *α* represents the expansivity [(*∂V*/*∂T)_P_*/*V*_0_] and *β* the compressibility [−*(∂V*/*∂T)_T_*/*V_0_*].

The lattice energy, *E*, is taken to be

$$E=\left(-\delta e^{2}/r-C/r^{6}-D/r^{8}+Be^{-r/\rho }\right)N$$ (3)

and the first two derivatives are given by

$${\left(\partial E/\partial V\right)}_{T}=\left[\delta e^{2}/r+6C/r^{6}+8D/r^{8}-Bre^{-r/\rho }/\rho \right]N/3V$$ (4)

and

$${\left(\partial^{2}E/\partial V^{2}\right)}_{T}=\left[4\delta e^{2}/r+54C/r^{6}+88D/r^{8}-Br\left(2+r/\rho \right)e^{-r}{/}^{\rho }/\rho \right]N/9V^{2}$$ (5)

where *δ* is the Madelung constant, *r*, the nearest neighbor distance $\left(r_{\text{fcc}}=a_{0}/2;\;r_{\text{sc}}=a_{0}\frac{\sqrt{3}}{2}\right)$, *e*, the electronic charge (4.80×10^−10^ esu), *C* and *D*, Van der Waals interaction coefficients, *B* and *ρ*, the repulsive parameters, and *N*, Avogadro’s number.

Equations 1 and 4 and eqs 2 and 5 are set equal for each phase at the transition point and the systems of two equations may be solved for the parameters *B* and *ρ* applicable to each phase. In practice, the equations are solved for *ρ* and *Be*^−^*^r^*^/^*^ρ^* rather than for the parameters^2^ *B* and *ρ*.

### 4.2. Data Used for Calculations

Experimental data used in the calculations were taken almost exclusively from the reports of Bridgman and differ somewhat from the data used by Tosi and Fumi [3]. Numerical values used are tabulated in table 2 with the sources being identified by the footnotes. Values enclosed in parentheses represent estimates for which experimental data were not available. Such estimates may be subject to errors exceeding the usual experimental uncertainties. The effect of errors on the calculated lattice energies will be discussed later.

### 4.3. Lattice Energies

Calculated values of *ρ* and *Be*^−^*^r^*^/^*^ρ^* for the two structures at the transition pressure are given in table 4. The lattice energies of both structures obtained from eq 3 using the data of table 2 and the values of *Be*^−^*^r^*^/^*^ρ^* from table 4 are given in cols 6 and 7 of table 4. Column 8 of table 4 gives the *P*Δ*V* term for the structure change calculated from the lattice parameters rather than from Bridgman’s data [6].

Although the numerical results of table 4 differ slightly from the corresponding values obtained by Tosi and Fumi [3] the agreement is encouraging because of the difference in the data used in the two calculations. It will be noted that the repulsive parameter, *ρ*, is generally smaller in the more compact CsCl type structure. This is in agreement with the findings of Tosi and Fumi [3], who observed that the experimental evidence for a smaller compressibility in the sc form required a smaller value for p. If the Born-Mayer treatment is adequate, then *E*_fcc_+*P*Δ*V*−*E*_sc_=0, provided the latent heat of the transition is negligible. The present data are less consistent with this requirement than the results of Tosi and Fumi [3]. Therefore, it is necessary to evaluate the latent heat and the effect of the experimental errors on the results.

### 4.4. Latent Heat of Transition

The anticipated agreement between lattice energies of the two structures when allowance is made for the work done at transition is predicated on the assumption that the entropy change at the transition is negligible. Although plausible arguments can be advanced to support this thesis [3], the experimental evidence has not been examined in detail. Jacobs [9] concluded that the latent heat could be neglected on the basis of Bridgman’s data [2, 3] but, on close examination, Bridgman’s results do not appear to be convincing. For example, Bridgman [4, 5] reported *dT*/*dP* positive for RbI, KI, and KCl but negative for RbBr, RbCl, and KBr and noted [4] that the accuracy of his data on the variation of transition pressure with temperature was questionable. Inspection of Bridgman’s data shows that the inaccuracy arises principally from the fact that the transitions are sluggish; and the transition pressures cannot be located with precision. In the rubidium halides there appears to be a region of indifference of about 1,000 kg/cm^2^. Although his data and figures for the potassium halides do not indicate this region, Bridgman reported [5] that the region of indifference was about 13,000 kg/cm^2^ for both KBr and KI.

Recently Clark [22] studied the effect of pressure on the melting points of some of the alkali halides and observed the triple points between the liquid phase and the two crystalline forms of CsCl, RbCl, and KCl. On the basis of his data *dT*/*dP*>0 for CsCl and he obtained a latent heat of 0.05×10^−12^ erg for the transition between the two crystalline forms. Combining his triple point data with Bridgman's data at lower temperatures indicates that *dT*/*dP*>*0* for RbCl but *dT*/*dP*<*0* for KCl. Assuming *dT*/*dP* independent of *P* and T, the latent heat for the transition in RbCl is estimated to be not more than 0.01×10^−12^ erg. For KCl the latent heat is negative but even smaller in magnitude. From the similarities in the properties of the alkali halides, it seems most probable that the latent heats for the other halides can be neglected on the scale of energies of interest here.

### 4.5. Effect of Errors on the Lattice Energies

As the entropy change at the transition appears to be negligible, the quantity *E*_fcc_+*P*Δ*V*−*E_sc_* should be zero for all the halides studied, provided the theory is adequate and the data used in the calculation are sufficiently precise. It is of interest to inquire whether, in view of the uncertainties in the data used for the calculation, the quantity E_fcc_+PΔV−*E_sc_* is significantly different from zero.

To obtain the answer to this question the calculation of the lattice energies was analyzed by the law of propagation of errors and an estimate of the reliability of the lattice energies was obtained. The method of calculation and the results are given in the appendix. The standard deviations of the lattice energies given in table *5* of the appendix were used to evaluate the significance of the deviations of the energies from zero by investigating the ratio $\left|E_{\text{fcc}}+P\Delta V-E_{\text{sc}}\right|/\sqrt{\sigma_{\text{fcc}}^{2}+\sigma_{\text{sc}}^{2}+\sigma_{P\Delta V}^{2}}$. All values necessary for the evaluation are given in the tables except those for $\sigma_{P\Delta V}^{2}$ which are insignificant in comparison with $\sigma_{\text{fcc}}^{2}$ and $\sigma_{\text{sc}}^{2}$.

If the rule is adopted to consider the deviations to be significantly different from zero only when such deviations exceed twice the respective standard deviations calculated, then only values for KI and CsF exceed these limits. From the fact that all other values are not significant in this sense it would appear that two possibilities exist, first the latent heats of transition in these two instances are not negligible, or second that the estimates of the experimental errors were too low. Of these two the latter seems much more probable. Some discrepancies in the data on KI were noted earlier and the assumed error was not sufficient to encompass such data in the calculations. It is by no means clear, as noted earlier, that Bridgman's data on CsF can be accepted as applying to the anhydrous salt. With these facts in mind it appears justified to conclude that, within the experimental error of the available data, the Born-Mayer treatment with the modifications introduced by Tosi and Fumi produces a satisfactory representation of the energies of the high-pressure forms of the alkali halides.

## Figures and Tables

**Table 1:** X-ray diffraction data on the alkali halides at high pressure*s*

Index	KI (P≈19 kb)		KBr (P≈22 kb)		KCl (P≈22 kb)		KF (P≈35kb)		RbI (P≈4.5 kb)		RbBr (P≈5kb)		RbCl (P≈5kb)		RbF (P≈12kb)		CsF (P≈48 kb)	
	*d*	a	*d*	a	*d*	a	*d*	a	*d*	a	*d*	a	*d*	a	*d*	a	*d*	a
																		
	*cm*^−8^	*cm*^−8^	*cm*^−8^	*cm*^−8^	*cm*^−8^	*cm*^−8^	*cm*^−8^	*cm*^−8^	*cm*^−8^	*cm*^−8^	*cm*^−8^	*cm*^−8^	*cm*^−8^	*cm*^−8^	*cm*^−8^	*cm*^−8^	*cm*^−8^	*cm*^−8^
100	3.920	3.920	……	……	……	……	3.076	3.076	……	……	……	……	3.876	3.876	3.300	3.300	3.370	3.370
(200 fcc)	3.316	fcc	3.206	fee	3.081	fcc	2.667	fee	3.621	fcc	3.410	fee	3.263	fcc	2.826	fcc	2.995	fcc
110	2.783	3.936	2.647	3.743	2.533	3.582	……	……	3.090	4.370	2.881	4.074	2.757	3.898	2.315	3.274	2.394	3.386
(220 fcc)	2.382	fcc	……	……	……	……	……	……	2.590	fcc	2.408	fcc	2.318	fcc	1.989	fcc	……	……
111	2.254	3.904	……	……	……	……	2.158	3.052	……	……	……	……	……	……	……	……	1.978	3.426
200	1.982	3.964	1.875	3.750	……	……	……	……	2.176	4.350	2.038	4.076	1.964	3.928	1.650	3.300	1.697	3.394
210	1.770	3.958	……	……	……	……	……	……	……	……	……	……	……	……	……	……	1.518	3.394
211	1.614	3.953	1.528	3.743	1.458	3.571	……	……	1.771	4.338	1.674	4.100	1.602	3.923	……	……	1.378	3.375
(420 fcc)	……	……	……	……	……	……	……	……	……	……	1.528	fcc	……	……	……	……	……	……
220	1.396	3.948	……	……	……	……	……	……	1.527	4.320	1.452	4.107	……	……	……	……	……	……
Average	……	3.94	……	3.74	……	3.58	……	3.06	……	4.34	……	4.09	……	3.91	……	3.29	……	3.39

**Table 2:** Numerical data used for lattice energy calculations

Halide	r at transition		C^a^		D^a^		P at transition^b^	*β* at transition^c^		*β* at 1 bar^d^	*α* at 1 bar^e^	(*∂β*/*∂*T)_P_ at 1 bar^d^	(*∂β*/*∂*P)_T_ at 1 bar^d^
								
	fcc	sc	fcc	sc	fcc	sc	10^9^ dyne/cm^2^	fcc	sc	fcc 10^−12^ cm^2^/dyne	fcc 10^−6^/deg	fcc 10^−15^ cm^2^/dyne deg	fcc 10^−22^ cm^4^/dyne^2^
			
	10^−8^ cm		10^−60^ erg cm^6^		10^−76^ erg cm^8^			10^−12^cm^2^/dyne					
									
KI	3.409	3.55	924	1472	1420	2516	17.85	6.6	5.2	8.54	125	5.12	3.3
KBr	3.203	3.23	605	930	800	1343	18.05	6.1	4.8	6.70	110	4.03	2.1
KCl	3.057	3.08	452	680	560	794	19.68	4.6	3.8	5.63	101	2.70	1.5
KF	2.601	2.76	167	246	150	249	(20.0)	2.2	(1.5)	3.31	100	0.40	0.66
RbI	3.628	3.76	1330	2039	2240	3675	3.97	8.5	7.1	9.58	119	6.52	4.1
RbBr	3.406	3.57	898	1346	1340	2085	4.51	7.2	5.1	7.94	104	(5.30) f	2.8
RbCl	3.257	3.39	691	1023	960	1267	4.90	6.1	4.9	6.70	98	5.30	(1.6)
RbF	2.783	2.84	278	409	290	442	(11.76)	(3.0)	(2.4)	(3.90)	(90)	(4.00)	(0.80)
CsF	2.943	3.04	495	1450	600	2390	(20.0)	2.5	(1.5)	4.30	95	4.40	1.2

a Values taken from ref. 20 or calculated from data of ref. 21.

b See ref. 6; values in parentheses estimated in these experiments.

c Calculated from data of ref. 4 and 8.

d See ref. 8 and 18.

e See ref. 18 and 19.

f This value is estimated. The value given in ref. 8 appears to be out of line with all the other values.

**Table 3:** Volume change at the transition (fcc→sc)

−Δ*V*/*V*_fcc_×100		
Halide	Present work	P–V data (Bridgman [5])
		
KI	22.2	8.5
KBr	19.3	10.5
KCl	18.3	11.2
KF	10.8	…
RbI	13.9	10. 7
RbBr	13.4	11.3
RbCl	13.4	14.6
RbF	17.3	…
CsF	10.0	…

**Table 4:** Repulsive parameters and lattice energies of alkali halides at transition points

Halide	*ρ*, 10^−8^ cm		Be^−r/^*^ρ^*, 10^−12^ erg		Lattice energy, 10^−12^ erg		PΔV at transition, 10^−12^ erg
				
	*fcc*	*sc*	*fcc*	*sc*	*fcc*	*sc*	
KI	0.417	0.409	2.34	2.51	−10.15	−10.41	0.31
KBr	.415	.401	2.48	2.47	−10.74	−10.88	.23
KCl	.369	.370	2.36	2.39	−11.45	−11.47	.21
KF	.308	.289	2.42	2.06	−13.69	−13.48	.06
RbI	.351	.354	1.49	1.49	−10.29	−10.10	.05
RbBr	.344	.305	1.62	1.41	−10.86	−10.81	.05
RbCl	.307	.293	1.56	1.44	−11.47	−11.30	.05
RbF	.250	.244	1.74	1.73	−13.43	−13.40	.09
CsF	.210	.189	1.52	1.74	−13.05	−13.80	.22

**Table 5:** Individual variance terms in error calculations

Halide	${\left(\frac{\partial E}{\partial T}\right)}^{2}\sigma_{T}{\;}^{2}$	${\left(\frac{\partial E}{\partial c}\right)}^{2}\sigma_{C}{\;}^{2}$	${\left(\frac{\partial E}{\partial D}\right)}^{2}\sigma_{D}{\;}^{2}$	${\left(\frac{\partial E}{\partial \alpha }\right)}^{2}\sigma_{\alpha }{\;}^{2}$	${\left(\frac{\partial E}{\partial \beta }\right)}^{2}\sigma_{\beta }{\;}^{2}$	${\left(\frac{\partial E}{\partial P}\right)}^{2}\sigma_{P}{\;}^{2}$	${\left(\frac{\partial E}{\partial r}\right)}^{2}\sigma_{r}{\;}^{2}$	${\left(\frac{\partial E}{\partial \frac{\partial \beta }{\partial T}}\right)}^{2}\sigma_{\frac{\partial \beta }{\partial T}}{\;}^{2}$	${\left(\frac{\partial E}{\partial \frac{\partial \beta }{\partial P}}\right)}^{2}\sigma_{\frac{\partial \beta }{\partial P}}{\;}^{2}$	*σ_E_*^2^	$\sqrt{\sigma_{E}{\;}^{2}}/N$
	(±5° C)	(±10%)	(±10%)	*	(±10%)	**	***	(±25%)	(±25%)	†	
	10^17^ erg^2^	10^18^ erg^2^	10^16^ erg^2^	10^16^ erg^2^	10^20^ erg^2^	10^20^ erg^2^	10^20^ erg^2^	10^20^ erg^2^	10^20^ erg^2^	10^20^ erg^2^	10^−12^ erg

fcc											

KI	3	42	33	4	1	15	86	0	0	102	0.17
KBr	6	8	44	4	3	21	140	1	0	164	0.21
KCl	5	18	6	4	6	15	155	1	1	178	0.22
KF	20	2	39	5	33	6	253	0	1	293	0.28
RbI	11	14	6	5	6	0	107	1	1	116	0.18
RbBr	7	25	5	5	9	0	136	1	1	148	0.20
RbCl	16	7	6	5	13	0	139	1	0	154	0.21
RbF	5	15	0	5	52	0	210	3	3	268	0.27
CsF	4	116	124	1	1	3	126	0	0	132	0.19

sc											

KI	4	36	25	78	4	17	153	0	0	176	0.22
KBr	4	24	13	67	6	17	2	1	0	26	0.08
KCl	4	6	14	80	10	11	2	1	1	25	0.08
KF	26	12	81	114	7	5	2	1	27	42	0.11
RbI	8	19	13	90	8	0	28	1	1	38	0.10
RbBr	7	25	8	74	14	0	7	1	1	23	0.08
RbCl	13	21	36	60	16	0	31	1	0	49	0.12
RbF	12	46	64	66	77	0	226	2	3	309	0.29
CsF	11	324	4	48	29	5	32	4	2	74	0.14

* For the fcc structures the range of *α* was taken to be ±10%, for the sc structures ±25%.

** For the potassium halides the range of *P* was taken to be ±5 kb; for the rubidium halides, ±0.5 kb; for CsF, ±5 kb.

*** For the fcc structures the range of *r* was taken to be 0.1A; for the sc structures the range was obtained from table 1.

† The data of the table have all been rounded off. The sum *σ_E_*^2^ was obtained from the unrounded data and subsequently rounded. Therefore, the values given for *σ_E_*^2^ may differ from the sums of the tabulated terms.

## Footnotes

1 Figures in brackets indicate the literature references at the end of this paper.

2 From the manner in which the equations are formulated it follows that the parameters and energies are determined for an ion pair. These units will be used throughout. Thus the energy in ergs will be understood to be ergs/ion pair.

## 5. Appendix

Given a quantity *E* which is an explicit function of variables *x*_1_, *x*_2_ *… x_n_* each of which is a function of the quantities *y*_1_,*y*_2_ *…y_n_*, i.e.,

$$\begin{array}{l} E=f\left(x_{1}x_{2}\ldots x_{n}\right)\;\text{with}\;x_{1}=\varphi \left(y_{1}y_{2}\ldots y_{n}\right),\\ x_{2}=\psi \left(y_{1}y_{2}\ldots y_{n}\right)\;\ldots \;x_{n}=\xi \left(y_{1}y_{2}\ldots y_{n}\right). \end{array}$$ (1)

Then the variance of *E*, $\sigma_{E}^{2}$, can be expressed to a first approximation in terms of the variances of the quantities *y*_1_,*y*_2_ *…y_n_*, $\left[\sigma_{y_{1}}^{2},\sigma_{y_{2}}^{2}\ldots \text{etc}.\right]$, which are assumed to be statistically independent, as follows:

$$\sigma_{E}^{2}≃{\left(\frac{\partial E}{\partial y_{1}}\right)}^{2}\sigma_{y_{1}}^{2}+{\left(\frac{\partial E}{\partial y_{2}}\right)}^{2}\sigma_{y_{2}}^{2}+\ldots {\left(\frac{\partial E}{\partial y_{n}}\right)}^{2}\sigma_{y_{n}}^{2}$$ (2)

where

$$\frac{\partial E}{\partial y_{1}}=\frac{\partial E}{\partial x_{1}}\frac{\partial x_{1}}{\partial y_{1}}+\frac{\partial E}{\partial x_{2}}\frac{\partial x_{2}}{\partial y_{1}}+\cdots \frac{\partial E}{\partial x_{n}}\frac{\partial x_{n}}{\partial y_{1}}$$ (3)

$$\frac{\partial E}{\partial y_{2}}=\frac{\partial E}{\partial x_{1}}\frac{\partial x_{1}}{\partial y_{2}}+\frac{\partial E}{\partial x_{2}}\frac{\partial x_{2}}{\partial y_{2}}+\cdots \frac{\partial E}{\partial x_{n}}\frac{\partial x_{n}}{\partial y_{2}}$$ (4)

$$\begin{array}{cccc} \vdots & \vdots & \vdots & \vdots \\ \frac{\partial E}{\partial y_{n}}= & \frac{\partial E}{\partial x_{1}}\frac{\partial x_{1}}{\partial y_{n}}+ & \frac{\partial E}{\partial x_{2}}\frac{\partial x_{2}}{\partial y_{n}}+ & \cdots \frac{\partial E}{\partial x_{n}}\frac{\partial x_{n}}{\partial y_{n}} \end{array}$$ (5)

If the following definitions are made:

$$x=3y\left(\frac{T\alpha }{\beta }-P\right);$$ (6)

$$y=\left(\frac{\delta e^{2}}{r}+\frac{6C}{r^{6}}+\frac{8D}{r^{8}}\right)N;$$ (7)

$$z=-\left(\frac{4\delta e^{2}}{r}+\frac{54C}{r^{6}}+\frac{88D}{r^{8}}\right)N;$$ (8)

$$w=\left[1+\left\{{\left(\frac{\partial \beta }{\partial T}\right)}_{P}+{\left(\frac{\partial \beta }{\partial P}\right)}_{T}\frac{\alpha }{\beta }\right\}\frac{T}{\beta }\right]\frac{9V}{\beta };$$ (9)

then it can be shown that

$$NBe^{-r/\rho }={\left(y-x\right)}^{2}/\left[\left(w-z\right)-2\left(y-x\right)\right].$$ (10)

If we define

$$R=-\left(\frac{\delta e^{2}}{r}+\frac{C}{r^{6}}+\frac{D}{r^{8}}\right)N$$ (11)

then the lattice energy can be written

$$E=R+{\left(y-x\right)}^{2}/\left[\left(w-z\right)-2\left(y-x\right)\right].$$ (12)

The quantities *x*, *y*, *z*, *w*, and *R* are identified with the variables *x* of eqs 3–5 while each of the *y*_1_ … *y_n_* can be identified with one of the parameters *r*, *T*, *α*, (∂*β*/∂*T*)etc. in the defining eqs 6–9 and 11. Therefore each of the appropriate partial derivatives in eqs 3–5 can be computed and evaluated. To evaluate eq 2 numerical values must be assigned to the variances of each parameter. The extreme range of each parameter was estimated. These estimates were based in part on previous experience in measuring such quantities and in part on available data. The range was arbitrarily defined to be equal to 5*σ* and the appropriate values of *σ*^2^ calculated.

The results of the calculations are given in table 5 which gives values for the various terms of eq. 2 for both forms of each halide. Each column is headed by the term to which the data refer and the range assumed for the parameter involved. The range is shown in parenthesis where applicable to all data in the column or in the footnotes where variable ranges were used. Column 12 gives the square root of the sum of the individual terms divided by Avogadro’s number. The values in this column represent the standard deviations of the lattice energies given in table 4.

The ranges were assigned as follows:

- For a working temperature of 298° K, a variation of ±5° K appears to be the maximum expected under normal laboratory conditions.
- No reasonable estimates for variations in the values of *C* and *D* were apparent. Consequently a range of ±10 percent was assumed. From the results it appears that the term in *D* will be negligible regardless of the range assumed.
- Measured values of *α* are available for the fcc structures. Considering both experimental errors and the variation of *α* with *P*, a value of ± 10 percent was assumed for the fcc phases. No data are available for the sc structures and to compensate for a change in *α* with structure the range was increased to ±25 percent for the sc materials. The term is unimportant and much larger variations could be tolerated without affecting the total error.
- Measured values of *β* are available for most halides. The range of *β* was taken somewhat arbitrarily to be ±10 percent. With the present data this term may represent as much as 1/10 of *σ_E_*^2^. With better data for the other quantities better estimates for *β* will be necessary.
- The uncertainties in the derivatives of *β* with *P* and *T* were set at ±25 percent. Both quantities are small and might not be expected to be sensitive to *P* and structure. They contribute only a very small amount to *σ_E_*^2^ so that even if the variations were outside the limits assumed they would not affect the results appreciably.
- The limits on *P* correspond to the estimates of Bridgman [4, 5] of the region of indifference. For the potassium halides and CsF a range of ±5kb was assumed while for the rubidium halides ±0.5kb was taken. For a few of the sc structures this uncertainty in *P* is the direct cause of a large part of the uncertainty *(σ_E_*^2^) in the lattice energy. For all of the fcc and most of the sc structures its effect is subordinate to that of the unit cell size.
- The limits on *r* for the sc structures were obtained directly from the X-ray diffraction data given in table 1 and were taken to be the range of the values for *a*_0_. The limits on *r* for the fcc structures are more difficult to estimate and it was finally decided to assume a range of ±0.05Å. Despite the fact that this figure may be on the high side, it represents the maximum for the sc structures which were measured directly. Although Bridgman’s compression data which were used to calculate *r* at the transition point for the fcc phases are presumably more precise than the estimated range, it is believed that the inherent difficulties involved in measurements where both phases coexist over a wide pressure range makes the variation in *r* assumed here not unreasonable.
